# Quantitative PET imaging of PD-L1 expression in xenograft and syngeneic tumour models using a site-specifically labelled PD-L1 antibody

**DOI:** 10.1007/s00259-019-04646-4

**Published:** 2019-12-27

**Authors:** Camilla Christensen, Lotte K. Kristensen, Maria Z. Alfsen, Carsten H. Nielsen, Andreas Kjaer

**Affiliations:** 1 Minerva Imaging, Copenhagen, Denmark; 2 Department of Clinical Physiology, Nuclear Medicine & PET and Cluster for Molecular Imaging, Department of Biomedical Sciences, Rigshospitalet and University of Copenhagen, Copenhagen, Denmark

**Keywords:** Molecular imaging, Positron emission tomography (PET), PD-L1, Immunotherapy, Immune checkpoint inhibition

## Abstract

**Purpose:**

Despite remarkable clinical responses and prolonged survival across several cancers, not all patients benefit from PD-1/PD-L1 immune checkpoint blockade. Accordingly, assessment of tumour PD-L1 expression by immunohistochemistry (IHC) is increasingly applied to guide patient selection, therapeutic monitoring, and improve overall response rates. However, tissue-based methods are invasive and prone to sampling error. We therefore developed a PET radiotracer to specifically detect PD-L1 expression in a non-invasive manner, which could be of diagnostic and predictive value.

**Methods:**

Anti-PD-L1 (clone 6E11, Genentech) was site-specifically conjugated with DIBO-DFO and radiolabelled with ^89^Zr (^89^Zr-DFO-6E11). ^89^Zr-DFO-6E11 was optimized in vivo by longitudinal PET imaging and dose escalation with excess unlabelled 6E11 in HCC827 tumour-bearing mice. Specificity of ^89^Zr-DFO-6E11 was evaluated in NSCLC xenografts and syngeneic tumour models with different levels of PD-L1 expression. In vivo imaging data was supported by ex vivo biodistribution, flow cytometry, and IHC. To evaluate the predictive value of ^89^Zr-DFO-6E11 PET imaging, CT26 tumour-bearing mice were subjected to external radiation therapy (XRT) in combination with PD-L1 blockade.

**Results:**

^89^Zr-DFO-6E11 was successfully labelled with a high radiochemical purity. The HCC827 tumours and lymphoid tissue were identified by ^89^Zr-DFO-6E11 PET imaging, and co-injection with 6E11 increased the relative tumour uptake and decreased the splenic uptake. ^89^Zr-DFO-6E11 detected the differences in PD-L1 expression among tumour models as evaluated by ex vivo methods. ^89^Zr-DFO-6E11 quantified the increase in PD-L1 expression in tumours and spleens of irradiated mice. XRT and anti-PD-L1 therapy effectively inhibited tumour growth in CT26 tumour-bearing mice (*p* < 0.01), and the maximum ^89^Zr-DFO-6E11 tumour-to-muscle ratio correlated with response to therapy (*p* = 0.0252).

**Conclusion:**

PET imaging with ^89^Zr-DFO-6E11 is an attractive approach for specific, non-invasive, whole-body visualization of PD-L1 expression. PD-L1 expression can be modulated by radiotherapy regimens and ^89^Zr-DFO-6E11 PET is able to monitor these changes and predict the response to therapy in an immunocompetent tumour model.

**Electronic supplementary material:**

The online version of this article (10.1007/s00259-019-04646-4) contains supplementary material, which is available to authorized users.

## Introduction

Immune checkpoint therapy has recently emerged as an effective way of evading the immunosuppressive tumour microenvironment thus allowing the immune system to eradicate tumours. One of the major checkpoints probed for therapy is programmed cell death protein 1 (PD-1), most prominently expressed on the surface of T cells, B cells, and natural killer cells, and its inducible ligand programmed death ligand 1 (PD-L1) naturally expressed on a variety of cell types including some tumour cells, hepatocytes, muscle cells, epithelium and antigen-presenting cells [1]. To date, PD-1/PD-L1 antibodies have been approved for treatment of several cancers including melanoma, renal cell carcinoma, non-small cell lung cancer (NSCLC) and bladder cancer—and the list is rapidly expanding [2]. Durable, clinical responses and long-term survival across several tumour types have anchored the clinical utility of immune checkpoint therapy. However, in many cancers, the response rates are not impressive with a large proportion of non-responding patients.

Therefore, precise methods to reliably identify patients most likely to benefit from immune checkpoint inhibitors are needed as they have the potential to improve the overall response rates. Existing companion diagnostics to select patients eligible for anti-PD-L1 therapy include ex vivo assessment of PD-L1 expression in tumours by immunohistochemistry (IHC) [3, 4]. However, patient biopsies are randomly sampled and often poorly reflect the intra-tumour heterogeneity, temporal dynamics, and prospective metastasis. Moreover, responding patients with PD-L1 negative tumours as well as non-responding patients with PD-L1 positive tumours have been reported during the course of anti-PD-L1 therapy [5, 6]. Adding to this, assessing responses to immunotherapy by standard RECIST criteria may be challenging compared with conventional chemotherapy as tumour cells are not killed directly and the fact that pseudo progression often is observed in patients receiving immune checkpoint inhibitors [7–9].

Molecular imaging with positron emission tomography (PET) has the potential to overcome some of these challenges by allowing a more comprehensive look at the entire tumour and tumour burden including metastases in vivo. Further, the non-invasive and quantitative nature of PET combined with high sensitivity allows for rapid assessment of unique biomarkers of response potentially guiding therapy decisions. Consequently, progress has been made in PET imaging of the PD-1/PD-L1 axis. Full-length antibody-based PET tracers specific for human, mouse as well as humanized cross-reactive to mouse PD-L1 have been developed [10–12]. In particular, the clinical efficacy of atezolizumab in NSCLC, melanoma, bladder cancer, and renal cell carcinoma has gained attention and concurrently prompted the development of atezolizumab-based tracers preclinically as well as clinically [13–17].

While the majority of these studies have demonstrated specific PD-L1 imaging in syngeneic, xenograft, and humanized mouse tumour models, only a few has investigated the predictive value of pre-treatment immuno-PET imaging with atezolizumab [17]. In the present study, we radiolabelled a monoclonal antibody similar to atezolizumab (clone 6E11), a humanized PD-L1 antibody cross-reactive to mouse PD-L1, with zirconium-89 (^89^Zr). Given our experience with site-specific labelling by glycan modification [18], 6E11 was conjugated site-specifically and evaluated in NSCLC xenografts and syngeneic mouse tumour models with varying PD-L1 expression. Lastly, the utility of ^89^Zr-DFO-6E11 as an in vivo biomarker of response to anti-PD-L1 therapy was evaluated in immunocompetent mice.

## Materials and methods

### Cell culture and animal models

Human NSCLC cancer cells H1703 (ATCC CRL- 5889), H1993 (ATCC CRL-5909), HCC827 (ATCC, CRL-2868), murine colon carcinoma cells CT26.WT (ATCC, CRL-2638), and murine melanoma cells B16-F10 (ATCC, CRL-6475) were cultured according to standard procedures. All cell lines were obtained from LGC standards and tested negative for mycoplasma and a panel of murine pathogens.

H1703, H1993, and HCC827 cells were harvested in their exponential growth phase and resuspended at 1:1 in complete growth media and Matrigel™ (BD Biosciences) at a concentration of 50 × 10^6^ cells/mL. A 100-uL cell suspension (5 × 10^6^ cells/tumour) was injected subcutaneously into the flanks (1 tumour/mouse) above the hind limbs in 7-week-old female NMRI nude mice (Janvier Labs). CT26.WT and B16-F10 cells were harvested in their exponential growth phase and resuspended in PBS at a concentration of 0.3 × 10^6^ cells/mL. A 100-μL cell suspension (300,000 cells/tumour) was injected subcutaneously into the flank (1 tumour/mouse) above the hind limbs in 7-week-old female BALB/c (CT26.WT) or C57BL/6 (B16-F10) mice (Janvier Labs).

Tumours used for longitudinal imaging and biodistribution studies were grown until ~ 300 mm^3^ while tumours for the efficacy study were grown until ~ 100–150 mm^3^ prior to treatment.

### Antibody conjugation, radiolabelling, and stability measurements

Anti-PD-L1 (clone 6E11, Genentech) was site-specifically enzymatically modified on glycan chains or randomly conjugated to the desferrioxamine-p-benzyl-isothiocyanate (DFO-Bn-NCS, Macrocyclics) chelator according to previously established protocols [18]. GalT enzyme and UDP-GalNAz substrate for the site-specific modification were obtained from Thermo Fisher Scientific and endoglycosidase S2 was purchased from Genovis. The radiochemical purity was determined by radio-thin layer chromatography (radio-TLC) using an eluent of 50 mM EDTA (pH 5.5) on silica gel 60 TLC plates, where the antibody construct remains at the baseline, while ^89^Zr^4+^ ions and [^89^Zr]-EDTA elute with the solvent front. The level of aggregates was estimated by size exclusion-high-performance liquid chromatograph (SEC-HPLC) on a Yarra™ 3 μm SEC-3000 Column 150 × 7.8 mm (Phenomenex) with 0.1 M phosphate buffer pH 7 as mobile phase.

The degree of labelling (DOL) of site-specifically labelled ^89^Zr-DFO-6E11 was determined by mass spectrometry and the in vitro stability was evaluated up to 7 days after radiolabelling in either PBS (4 °C) or mouse plasma (37 °C) at a concentration of 1.7 MBq/mL. Samples were withdrawn up to 144 h after labelling. The radiochemical stability in buffer and plasma was determined by radio-TLC and SEC-HPLC as described above.

### Immuno-reactivity and saturation binding assay

The immuno-reactivity of ^89^Zr-DFO-6E11 was assessed according to the Lindmo assay [19]. Increasing concentrations of HCC827 cells (3.91 × 10^5^–5.0 × 10^7^ cells/mL) were incubated with 0.1-nM ^89^Zr-DFO-6E11 for 3 h at 4 °C. Cells were centrifuged at 500*g* for 5 min and the supernatants and pellets counted in a gamma counter (Wizard^2^, PerkinElmer). Cell-bound radioactivity was calculated as the ratio of cell-bound radioactivity to the total amount of added radioactivity.

The affinity of radiolabelled 6E11 was assessed by a saturation binding assay. HCC827 cells were harvested, added in triplicates (2 × 10^4^ cells) to a MultiScreenHTS BV Filter Plate 1.2 μm (#MSBVN1250, Merck Millipore) and washed twice in PBS. Eight different concentrations of ^89^Zr-DFO-6E11 (range 30 nM–0.01 nM) in PBS supplemented with 1% bovine serum albumin (BSA) were added into the wells. A parallel series was prepared containing 100-fold excess unlabelled 6E11 to assess non-specific binding. The plate was incubated for 4 h at 4 °C. After incubation, the plate was washed 3 times in PBS with 1% BSA using a vacuum manifold (Macherey-Nagel, Fisher Scientific). The plastic cover was removed from the plate bottom and the plate dried in a heat cabinet. The dry filters were transferred to counting tubes and counted in a gamma counter.

### Flow cytometry of PD-L1 expression

The surface expression of PD-L1 was evaluated by flow cytometry of H1703, H1993, HCC827, CT26, and B16F10 cell cultures. Cells were harvested, washed in FACS buffer (PBS without Ca^2+^ and Mg^2+^, 1% BSA, 0.5 mM EDTA, 0.1% NaN_3_) and resuspended at 1 × 10^6^ cells/mL. Human cell lines (H1703, H1993, HCC827) were incubated with anti-human PD-L1 antibody (#ab205921, Abcam) for 1 h at 4 °C, washed and stained for 30 min at 4 °C with AF488-anti-human IgG (#A11013, Life Technologies). Murine cell lines (CT26, B16F10) were incubated with anti-murine PD-L1 antibody (PE, #551892, BD) for 1 h at 4 °C. Cell-associated fluorescent intensity was quantified using FACSCanto II (BD Biosciences) and data analyzed using FlowJo Software (v10.0.7, Tree Star Inc.).

### Optimization of antibody dose

The optimal protein dose for PET imaging was investigated by an ex vivo biodistribution study with randomly labelled ^89^Zr-DFO-6E11. HCC827 tumour-bearing mice were randomized into five groups (*N* = 3/group) and injected immediately after end-of-synthesis (EOS) via the tail vein with 1.92 ± 0.02 (range 1.78–2.08) MBq ^89^Zr-DFO-6E11 diluted in 0.9% sterile NaCl prior to injection (200 μL total volume). The protein dose was 2.17 ± 0.03 (range 2.01–2.35) μg. Mice were co-dosed with 0, 10, 30, 100, or 500 μg of unlabelled 6E11. Blood was withdrawn by cardiac puncture and mice were euthanized 144 h after injection. Tumours and organs were resected, weighed, and the radioactivity counted in a gamma counter.

### Small animal PET/CT imaging

The optimal imaging time-point was assessed by longitudinal small animal PET/CT imaging in HCC827 tumour-bearing mice (*N* = 8). Mice were injected intravenously immediately after EOS through the tail vein with 1.89 ± 0.02 (range 1.78–2.01) MBq site-specifically labelled ^89^Zr-DFO-6E11 and 30 μg of unlabelled 6E11. Mice were anesthetized with sevoflurane (4% in 65% N_2_, 35% O_2_) and subjected to PET/CT imaging on an Inveon Multimodality PET/CT scanner (Siemens) 4, 24, 72, and 144 h post-injection (300, 300, 600, and 1200 s PET acquisition time, respectively).

Following optimization of imaging time-point, specificity of ^89^Zr-DFO-6E11 was evaluated in HCC827, H1993, and H1703 xenograft mouse models (*N* = 7/model) as well as CT26 and B16F10 syngeneic mouse models (*N* = 6/model). Mice were injected intravenously immediately after EOS with 1.2 ± 0.09 (range 0.65–2.82) MBq. PET/CT imaging was conducted 72 h post-injection (600 s PET acquisition time).

All images were reconstructed using a 3D maximum a posteriori algorithm with CT-based attenuation correction. Image analysis (Inveon Software, Siemens) was performed by drawing CT-based region of interests (ROIs). The uptake of ^89^Zr-DFO-6E11 was quantified as percent injected dose per gram tissue (%ID/g) assuming a tissue density of 1 g/cm^3^.

### Ex vivo biodistribution

A subgroup of mice (*N* = 3/model) was subjected to conventional ex vivo biodistribution after the last imaging session. Mice were euthanized, tumours and organs were resected, weighed, and the radioactivity counted in a gamma counter. Following counting, tumours were fixed in 4% paraformaldehyde for 24 h followed by transfer to 70% ethanol for paraffin embedding.

### Therapy

CT26 tumour-bearing mice were randomized into 4 treatment arms: control, external radiation therapy (XRT), XRT + anti-PD-L1, and anti-PD-L1 (*N* = 8/group). Mice from the XRT and the XRT + anti-PD-L1 group were placed in the radiation chamber in a restrainer allowing total fixation of the leg and the body was covered by lead shielding so that only the tumour was exposed to radiation. Two gray (Gy) were dosed at a rate of 1 Gy/min (320 kV, 12.5 mA) using a small animal irradiator (XRAD-320, pXi, CT) for three consecutive days. After the last radiation dose, mice from all groups were injected with 1.12 ± 0.11 MBq ^89^Zr-DFO-6E11 + 30 μg 6E11 intravenously and subjected to PET/CT imaging 72 h post-injection according to the above described protocol. Maximum tumour uptakes were calculated as a mean of the top 30% of hottest voxels. The tumour-to-muscle ratios were calculated as tumour(mean)/muscle(mean) and tumour(max)/muscle(mean). Following PET/CT imaging, subgroups of mice (XRT + anti-PD-L1 and anti-PD-L1 group) received 6 doses of 10 mg/kg anti-mouse PD-L1 (10F.9G2, #BE0101, BioXcell) every second or third day.

### Immunohistochemistry

Formalin-fixed tumours were embedded in paraffin, sectioned at 4 μm and mounted on SuperFrost ULTRA PLUS slides (Thermo Fisher Scientific). Sections were deparaffinized, rehydrated in a series of alcohols, and microwaved in citrate buffer pH = 6 for heat-induced epitope retrieval. Sections were blocked and stained with the following antibodies: anti-human PD-L1 antibody (#ab205921, Abcam) or anti-mouse PD-L1 (#ab238697, Abcam). Primary antibodies were detected using the EnVision + System-HRP labelled Polymer and Liquid DAB + substrate chromogen system (Agilent Technologies). All procedures were performed at room temperature and all tumours were stained in the same batch.

### Statistical analyses

Data are stated as mean ± SEM. One-way ANOVA with post hoc test corrected for multiple comparisons (Tukey) was applied to test for tumour volumes between groups (days 0 and 4), image contrast over the imaging time-course, and the tumour uptake values across xenograft and syngeneic models. Two-way ANOVA with repeated measures and Tukey’s multiple comparisons test was applied to compare tumour volumes over time. Survival was analyzed using the Kaplan–Meier method and the log-rank (Mantel–Cox test), where *p* < 0.008 was considered statistically significant when correcting for multiple comparisons using the Bonferroni method. *P* values ≤ 0.05 were considered statistically significant. Statistical analyses were performed using GraphPad Prism 8.0c (GraphPad Software).

## Results

### ^89^Zr-DFO-6E11 synthesis, stability, and in vitro characteristics

**Fig. 1:**
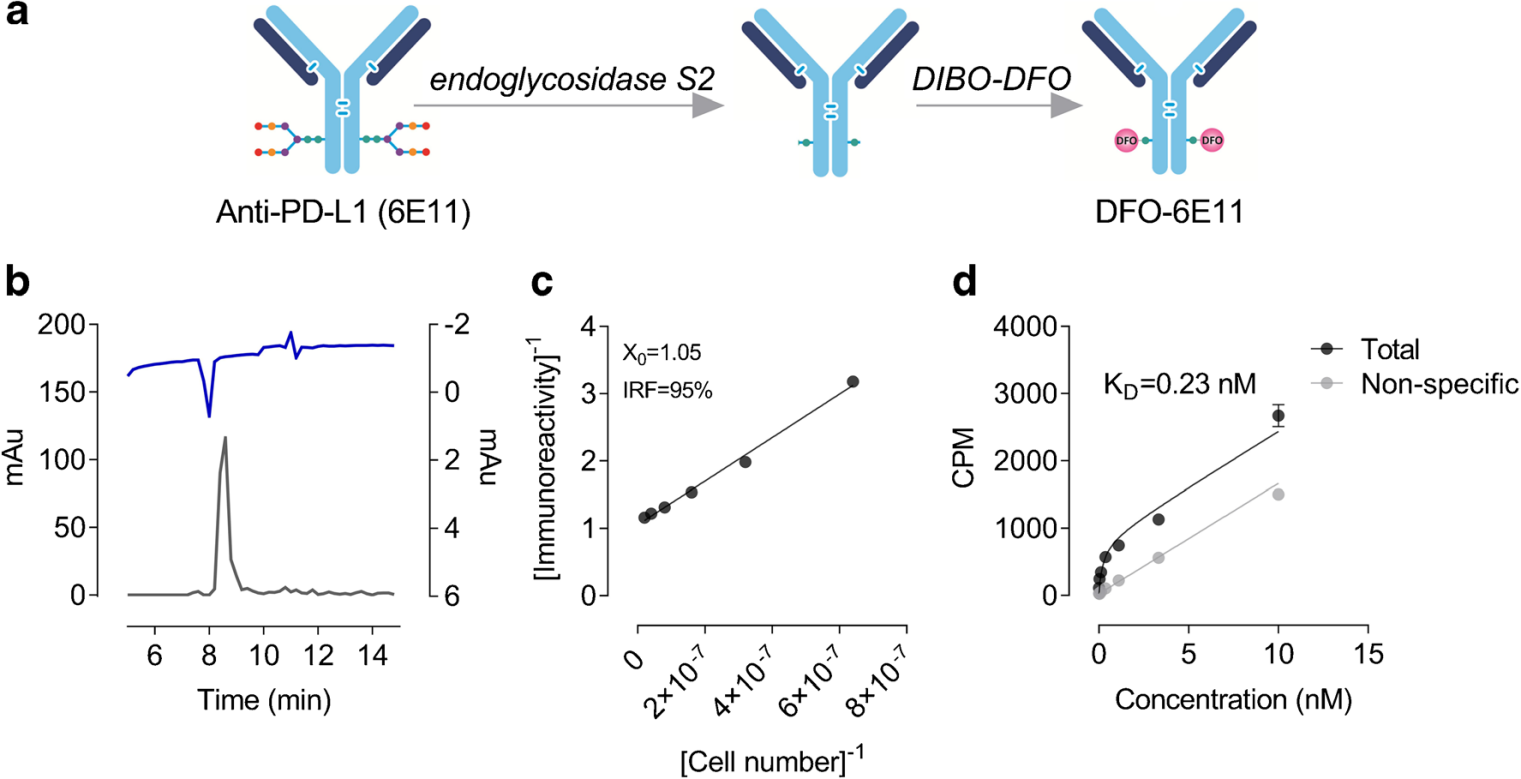
Tracer development and validation. **a** Graphical illustration of 6E11 chelator conjugation using endoglycosidase S2 and DIBO-DFO yielding 2 chelates per antibody on the heavy chain glycans. **b** HPLC chromatogram of ^89^Zr-DFO-6E11. **c** Immuno-reactivity assay of ^89^Zr-DFO-6E11 incubated with HCC827 cells (high PD-L1 expression). **d** Saturation binding assay of ^89^Zr-DFO-6E11 incubated with HCC827 cells. CPM, counts per minute; DIBO-DFO, dibenzocyclooctyne-desferrioxamine; *IRF*, immuno-reactive fraction; K_D_, dissociation constant

6E11 was successfully conjugated to DFO by site-specific modification (Fig. 1a) and labelled with ^89^Zr with a radiochemical yield of 19.4 ± 3.8 MBq. Radiochemical purity was > 99% as assessed by radio-TLC and aggregates were estimated to < 5% by SEC-HPLC. A representative chromatogram of ^89^Zr-DFO-6E11 is shown in Fig. 1b. The specific activity was 781.1 ± 140.5 MBq/mg and the DOL estimated to 2. Tracer specifications are listed in Table 1, where data for randomly conjugated 6E11 are included. ^89^Zr-DFO complex of ^89^Zr-DFO-6E11 was stable in buffer (> 99% intact) after 144 h of incubation as determined by radio-TLC. Plasma stability of ^89^Zr-DFO-6E11 as determined by SEC-HPLC showed 47% intact tracer after 144 h (Table S1).

**Table 1:** Specifications of ^89^Zr-DFO-6E11

	^89^Zr-DFO-6E11	
	Random	Site-specific
Radiochemical yield (MBq)	23.5 ± 6.4	19.4 ± 3.8
Purity HPLC (%)	> 95	> 95
Aggregates (%)	< 5	< 5
Purity radio-TLC (%)	> 99	> 99
Specific activity (MBq/mg)	896.0 ± 45.7	781.1 ± 140.5
Immuno-reactivity (%)	65	95
K_D_ (nM)	2.7	0.23
Degree of labelling (DOL)	–	2

Values are mean ± SEM

The immuno-reactive fraction was estimated to 95% at infinite antigen levels (Fig. 1c). ^89^Zr-DFO-6E11 exhibited affinity towards HCC827 (PD-L1 high expressing cells) in the nanomolar range with an estimated K_D_ of 0.23 nM (Fig. 1d).

### Optimization of antibody dose

A titration study with increasing concentrations of co-injected unlabelled 6E11 was performed in HCC827 tumour-bearing mice to determine the optimal antibody dose for ^89^Zr-DFO-6E11 imaging. The distribution of ^89^Zr-DFO-6E11 changed considerably with increasing dose with primary differences observed in terms of tumour, liver, and splenic uptake (Table 2).

**Table 2:** Effect of titration of antibody dose on ^89^Zr-DFO-6E11 uptake determined by ex vivo biodistribution 144 h post-injection in HCC827 tumour-bearing mice (*N* = 3/dose)

	+ 0 μg	+ 10 μg	+ 30 μg	+ 100 μg	+ 500 μg
Blood	0.04 ± 0.01	0.04 ± 0.01	0.04 ± 0.00	0.45 ± 0.29	1.86 ± 0.38
Bone	3.07 ± 0.08	3.15 ± 0.08	2.87 ± 0.24	2.74 ± 0.04	1.19 ± 0.52
Heart	1.01 ± 0.05	0.49 ± 0.05	0.50 ± 0.05	0.57 ± 0.07	0.83 ± 0.05
Intestine	0.45 ± 0.13	1.13 ± 0.18	0.90 ± 0.07	0.78 ± 0.11	0.57 ± 0.05
Kidney	2.11 ± 0.28	1.36 ± 0.04	1.34 ± 0.07	1.42 ± 0.07	1.37 ± 0.07
Liver	7.99 ± 0.03	3.54 ± 1.18	3.81 ± 0.37	3.51 ± 0.09	2.90 ± 0.11
Lungs	3.82 ± 0.59	1.39 ± 0.60	1.09 ± 0.11	1.20 ± 0.14	1.62 ± 0.02
Muscle	0.28 ± 0.04	0.16 ± 0.03	0.16 ± 0.01	0.21 ± 0.07	0.68 ± 0.47
Pancreas	0.35 ± 0.02	0.47 ± 0.05	0.44 ± 0.03	0.34 ± 0.06	0.46 ± 0.03
Spleen	14.44 ± 3.10	8.11 ± 2.41	5.59 ± 0.51	4.25 ± 0.35	3.36 ± 0.09
Stomach	0.26 ± 0.01	0.31 ± 0.02	0.38 ± 0.07	0.39 ± 0.02	0.39 ± 0.02
Tumour	0.35 ± 0.04	1.09 ± 0.39	1.72 ± 0.24	3.15 ± 0.55	3.07 ± 0.15

Values are mean ± SEM

The tumour uptake increased from 0.35 ± 0.04 %ID/g in mice receiving no co-dose to 3.07 ± 0.15 %ID/g in mice receiving a 500-μg co-dose, while the spleen uptake decreased from 14.44 ± 3.10 %ID/g in mice receiving no co-dose to 3.36 ± 0.09 %ID/g in mice receiving a 500-μg co-dose. The decrease in splenic uptake was also confirmed by the spleen-to-blood and spleen-to-muscle ratios (Table 3). The tumour-to-blood ratio increased from 9.01 ± 1.82 to 41.94 ± 1.84 with a 30-μg co-injection of unlabelled 6E11, while co-injection with 100 and 500 μg decreased the ratio further below the non-titrated dose uptake approximating blocking levels. To be noted is the distribution of ^89^Zr-DFO-6E11 in the liver with increasing dose. Liver uptake decreased from 7.99 ± 0.03 to 2.90 ± 0.11 with no and a 500-μg co-dose, respectively, confirming this site as primary clearance route. Based on these results, a co-injection of 30 μg of unlabelled 6E11 was applied in all further experimentation.

**Table 3:** Target-to-background ratios of ^*89*^Zr-DFO-6E11 determined by ex vivo biodistribution 144 h post-injection in HCC827 tumour-bearing mice (*N* = 3/dose)

	Tumour/blood	Tumour/muscle	Spleen/blood	Spleen/muscle
+ 0 μg	9.01 ± 1.82	1.25 ± 0.05	345.11 ± 34.65	56.61 ± 13.87
+ 10 μg	25.43 ± 4.00	7.28 ± 2.40	244.01 ± 100.9	47.26 ± 5.68
+ 30 μg	41.94 ± 1.84	10.74 ± 1.49	140.62 ± 15.58	34.69 ± 3.09
+ 100 μg	14.05 ± 4.70	17.23 ± 3.09	21.65 ± 8.47	24.75 ± 5.25
+ 500 μg	1.80 ± 0.33	10.34 ± 3.50	1.98 ± 0.37	11.20 ± 3.76

Values are mean ± SEM

### Longitudinal PET/CT imaging

**Fig. 2:**
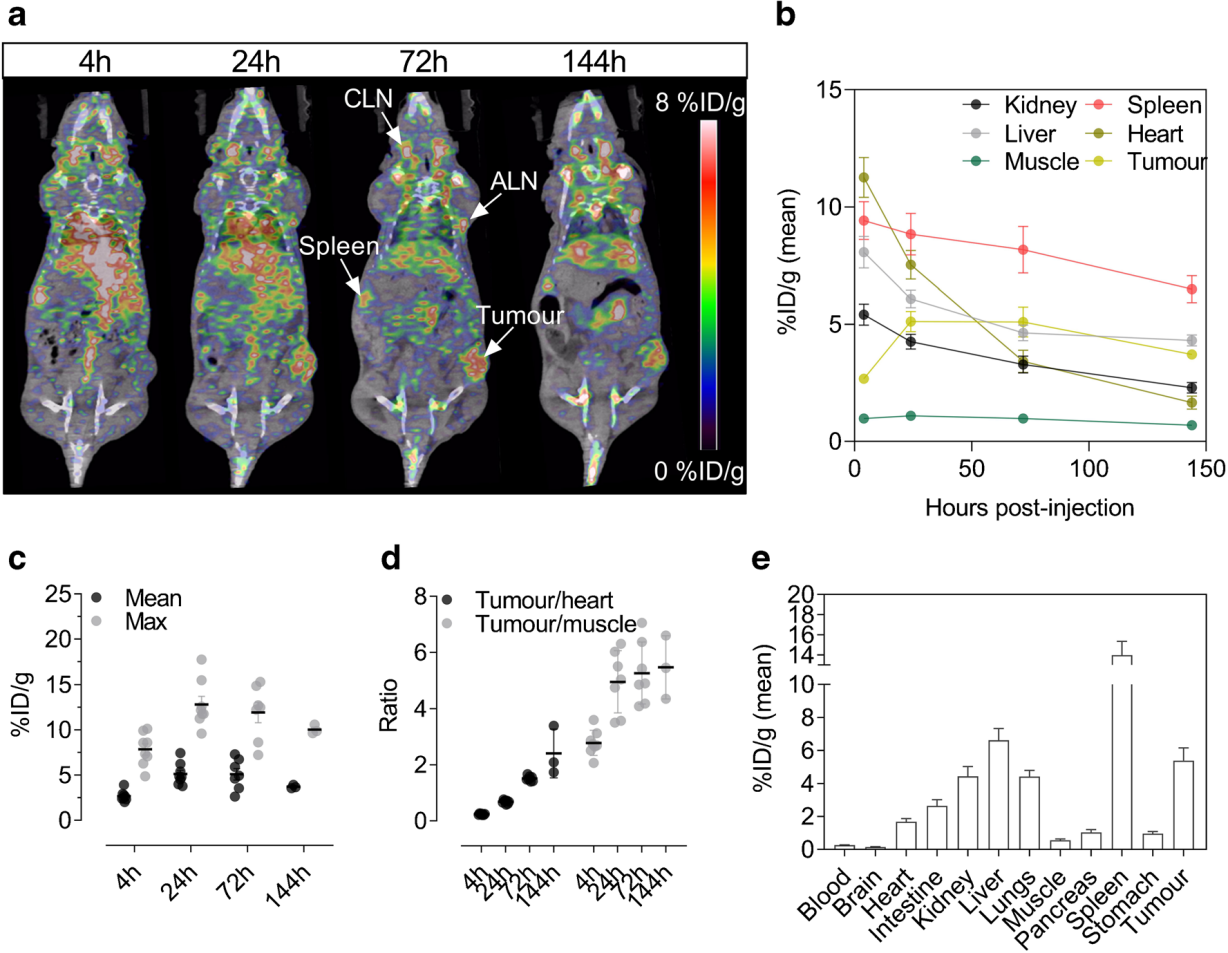
Small animal ^89^Zr-DFO-6E11 PET/CT in HCC827 tumour-bearing mice. **a** Representative PET/CT images of the same mouse at 4, 24, 72, and 144 h post-injection of ^89^Zr-DFO-6E11 illustrating targeting to lymphoid tissue and tumour. **b** Image-derived biodistribution over the imaging time-course in major organs. **c** Average (mean) and maximum (max) tumour uptake of ^89^Zr-DFO-6E11 expressed as %ID/g at 4, 24, 72, and 144 h post-injection. **d** Tumour-to-heart and tumour-to-muscle ratios of the mean ^89^Zr-DFO-6E11 uptake over the imaging time-course. **e** Ex vivo biodistribution measured by gamma counting of ^89^Zr-DFO-6E11 after the last imaging session 144 h post-injection. *N* = 8 for all time points except 144 h, where *N* = 3. Data are presented as mean ± SEM. %ID/g = % injected dose per gram tissue. ALN, axillary lymph node; CLN, cervical lymph node

The temporal in vivo distribution of ^89^Zr-DFO-6E11 was assessed by longitudinal PET/CT imaging in HCC827 tumour-bearing mice 4, 24, 72, and 144 h post-injection. Representative PET/CT images from the same mouse at each time point are illustrated in Fig. 2a and show targeting of ^89^Zr-DFO-6E11 to the HCC827 tumours and lymphoid tissue. The distribution in major organs is depicted in Fig. 2b and confirmed clearance of ^89^Zr-DFO-6E11 primarily through the hepatobiliary system.

The average tumour uptake was 2.7 ± 0.20, 5.12 ± 0.43, 5.1 ± 0.64, and 3.72 ± 0.11 %ID/g for the 4, 24, 72, and 144h time-point, respectively (Fig. 2c). Likewise, the maximum uptake within tumours was 7.9 ± 0.6, 12.8 ± 0.91, 11.9 ± 1.1, and 10.0 ± 0.28 %ID/g for the 4, 24, 72, and 144h time-point, respectively. No further improvement in tumour-to-muscle ratio was observed from 72 to 144 h (*p* = 0.9895) and 72 h post-injection was thus chosen as optimal imaging time-point (Fig. 2d). Ex vivo biodistribution after the last imaging time-point confirmed the PET data obtained in vivo with accumulation primarily seen in the liver, spleen, and tumour (Fig. 2e).

### Specificity of ^89^Zr-DFO-6E11

**Fig. 3:**
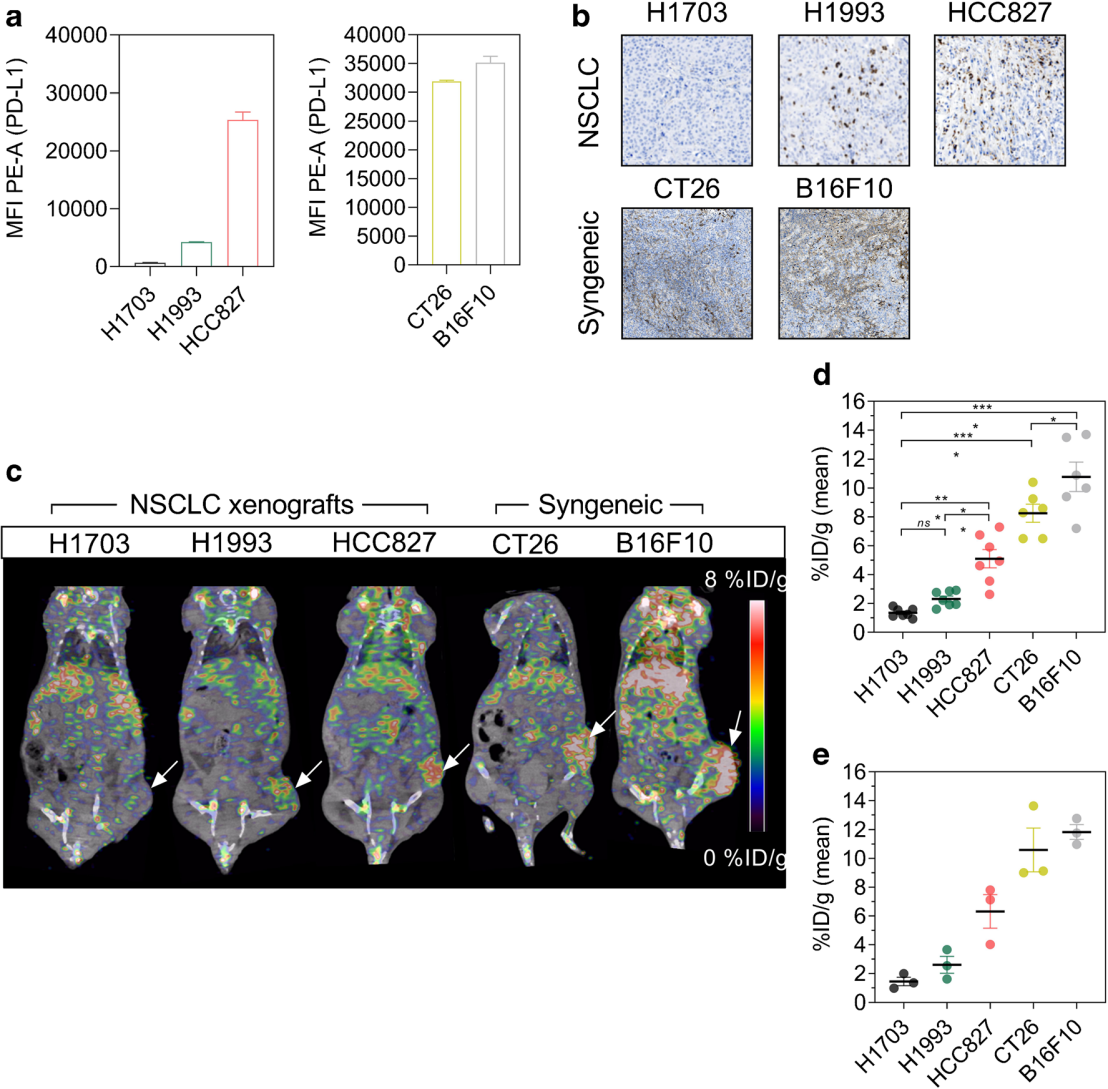
Specificity of ^89^Zr-DFO-6E11 in xenograft and syngeneic mouse tumour models. **a** Flow cytometric analysis of various cell lines for surface expression of PD-L1 using PE-conjugated anti-human or anti-mouse PD-L1 antibody. Data are presented as a mean of three independent experiments. **b** Ex vivo immunohistochemical staining of PD-L1 in NSCLC and syngeneic tumour models. **c** Representative PET/CT images of the H1703, H1993, HCC827 NSCLC xenografts, and CT26 and B16F10 syngeneic tumour models 72 h post-injection of ^89^Zr-DFO-6E11 illustrating targeting to lymphoid tissue and the tumour (indicated by arrows). **d** Mean tumour uptake of ^89^Zr-DFO-6E11 72 h post-injection quantified by PET ROI analysis and expressed as %ID/g (*N* = 7 for xenograft models, *N* = 6 for syngeneic models). **e** Ex vivo uptake in tumours of ^89^Zr-DFO-6E11 72 h post-injection of the different models measured by gamma counting (*N* = 3/model). Data are presented as mean ± SEM and the significance levels are indicated by asterisks (*). *p < 0.05, **p < 0.01, ***p < 0.001, ****p < 0.0001. %ID/g, percent injected dose per gram tissue; MFI, median fluorescent intensity; PE-A, phycoerythrin-area; NSCLC, non-small cell lung cancer

The ability of ^89^Zr-DFO-6E11 to image PD-L1 expression was evaluated in three NSCLC xenografts (H1703, H1993, and HC827) and two syngeneic models (CT26 and B16F10) with different PD-L1 expression levels. Initially, the expression level of all models was evaluated by flow cytometry, and data showed that H1703 could be characterized as low, H1993 as intermediate, and HCC827, CT26, and B16F10 as high PD-L1 expressing cell lines, relative to each other (Fig. 3a). The PD-L1 expression levels were further confirmed by IHC (Fig. 3b).

To evaluate the in vivo specificity of ^89^Zr-DFO-6E11, PET/CT imaging was applied to the same panel of tumour models and uptake quantified 72 h post-injection. ^89^Zr-DFO-6E11 detected the variation in PD-L1 expression as shown by the representative PET/CT images (Fig. 3c) and the mean in vivo tumour uptake across models thus confirming the cross-reactivity of 6E11 (Fig. 3d). The mean tumour uptake was 1.35 ± 0.1, 2.32 ± 0.2, 5.1 ± 0.6, 8.26 ± 0.6, and 10.78 ± 0.9 %ID/g for the H1703, H1993, HCC827, CT26, and B16F10 model, respectively. The uptake in HCC827 tumours was significantly different from the uptake in H1703 (*p* = 0.0003) and H1993 (*p* = 0.0075). Also, the uptake in CT26 and B16F10 tumours was significantly different (*p* = 0.0414) and were both significantly increased compared with H1703 (*p* < 0.0001). Overall, the uptake levels corresponded to the expression level shown by flow cytometry and IHC. Furthermore, gamma counting of tumours confirmed the uptake levels of ^89^Zr-DFO-6E11 measured by PET (Fig. 3e).

### Therapy-induced changes in PD-L1 expression detected with ^89^Zr-DFO-6E11

**Fig. 4:**
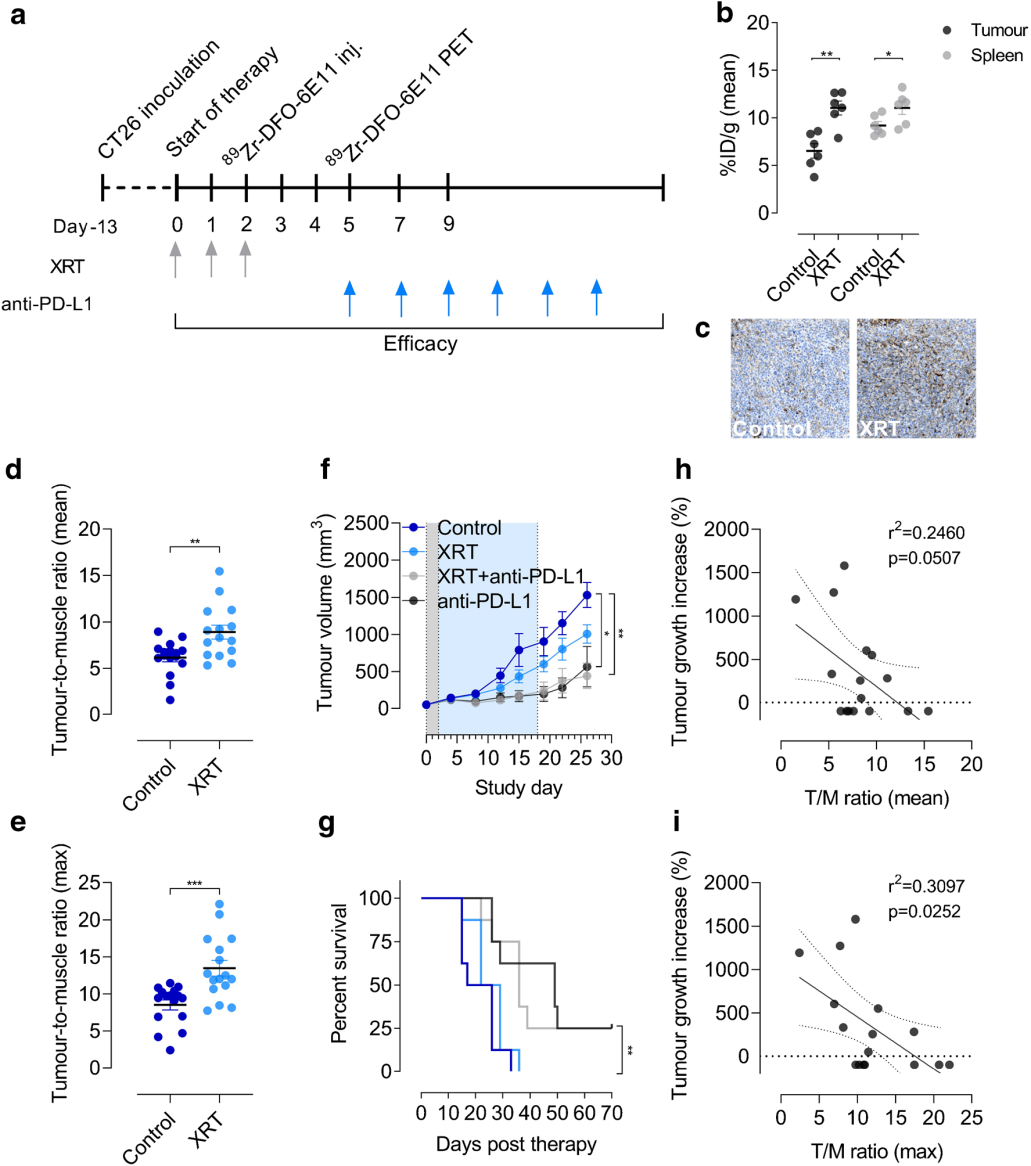
Treatment monitoring and prediction of anti-PD-L1 efficacy with ^89^Zr-DFO-6E11 in CT26 tumour-bearing immunocompetent mice. **a** Overview of timing of model establishment, ^89^Zr-DFO-6E11 injections, and therapy dosing (*N* = 8/group). Radiotherapy (XRT) was dosed 3 times (3 × 2 Gy) prior to ^89^Zr-DFO-6E11 injection and anti-mouse PD-L1 antibody was dosed 6 times over 2 weeks (10 mg/kg). **b** Mean ^89^Zr-DFO-6E11 uptake in tumours and spleens of control and XRT treated (3 × 2 Gy) mice 72 h post-injection of ^89^Zr-DFO-6E11 (*N* = 6/group). **c** Ex vivo immunohistochemical staining of PD-L1 in tumours of a control and XRT treated mouse. **d** Mean tumour-to-muscle ratio of the ^89^Zr-DFO-6E11 uptake in control and XRT treated mice (*N* = 16/group). **e** Maximum tumour-to-muscle ratio of the ^89^Zr-DFO-6E11 uptake in control and XRT treated mice (*N* = 16/group). **f** Tumour volume over time from the time of randomization (day 0) (*N* = 8/group). The gray area represents the XRT treatment period and the blue area the anti-PD-L1 treatment period. **g** Overall survival of mice in the different treatment groups (*N* = 8/group). **h** Tumour growth increase from day 4 to day 26 expressed as percent compared with the mean tumour-to-muscle (T/M) ratio of ^89^Zr-DFO-6E11 (*N* = 16). **i** Tumour growth increase from day 4 to day 26 expressed as percent compared with the maximum tumour-to-muscle (T/M) ratio of ^89^Zr-DFO-6E11 (*N* = 16). All uptake values are derived from PET ROI analysis and expressed as %ID/g. Data are presented as mean ± SEM and the significance levels are indicated by asterisks (*). *p < 0.05, **p < 0.01, ***p < 0.001, ****p < 0.0001. %ID/g, percent injected dose per gram tissue; XRT, external radiation therapy

Based on the numerous ongoing clinical efforts exploring the possible synergistic effects of immunotherapy combined with conventional cancer treatments, we designed an immunotherapy combination study with XRT and anti-PD-L1 therapy (Fig. 4a). Initially, we evaluated whether ^89^Zr-DFO-6E11 could detect the temporal dynamics of XRT-induced PD-L1 upregulation in a pilot study with CT26 tumour-bearing mice (Fig. 4b). Despite a fractionated radiation dose delivered directly to tumour the mean ^89^Zr-DFO-6E11 uptake was increased in both the tumour (*p* = 0.0076) and the spleen (*p* = 0.0100) of treated mice. Immunohistochemical staining of the same tumours confirmed an upregulation of PD-L1 expression and/or increased presence of PD-L1 positive cells (Fig. 4c).

For the immunotherapy combination study, mice were subjected to XRT, ^89^Zr-DFO-6E11 PET/CT, and concurrently dosed with anti-PD-L1. The mean (Fig. 4d, *p* = 0.0025) and maximum (Fig. 4e, *p* = 0.0005) tumour-to-muscle ratio of ^89^Zr-DFO-6E11 was increased in irradiated mice compared with control mice. Tumour growth was effectively inhibited in the anti-PD-L1 (*p* = 0.0476) and XRT + anti-PD-L1 (*p* = 0.0023) treated group compared with the control group on day 26 (last day of control group). No effect of XRT alone was observed (Fig. 4f*, p* = 0.1072). Similarly, overall survival was improved in the anti-PD-L1 (*p* = 0.0024) and XRT + anti-PD-L1 (*p* = 0.0023) treated mice compared with control mice (Fig. 4g).

### Increased tumour-to-muscle ratio of ^89^Zr-DFO-6E11 in responding mice

To investigate the overall association of ^89^Zr-DFO-6E11 tumour uptake and immunotherapeutic response to anti-PD-L1 therapy, the percent tumour growth increase at multiple days post-treatment initiation was calculated and plotted against the mean and maximum tumour-to-muscle ratios of ^89^Zr-DFO-6E11. The day prior to start of anti-PD-L1 therapy (day 4), there was no difference in tumour volumes between treatment groups (Fig. S1, *p* = 0.4731). Representative PET/CT images illustrating ROIs of the muscle and the tumour of a mouse are shown in Fig. S2. Percent tumour growth increase was calculated for day 15, 19, 22, and 26 and represents the time frame from when effect of therapy was evident from the tumour growth curves (day 15) and to the latest time-point sufficient numbers of mice were left in the control group (day 26). There was no association between percent tumour growth increase (day 26/4) and the mean tumour-to-muscle ratio in anti-PD-L1 treated mice (Fig. 4h, *r*^2^ = 0.2460, *p* = 0.0507). However, a significant negative correlation between percent tumour growth increase (day 26/4) and the maximum tumour-to-muscle ratio in anti-PD-L1 treated mice was found (Fig. 4i, *r*^2^ = 0.3097, *p* = 0.0252). Similarly, an association between percent tumour growth increase (day 15/4) and the maximum tumour-to-muscle ratio in anti-PD-L1 treated mice (Fig. S3b, *r*^2^ = 0.2973, *p* = 0.0289) was found. The recorded tumour growth on day 19 and 22 was not found to be associated with the ^89^Zr-DFO-6E11 tumour-to-muscle ratio (Fig. S3c–f).

## Discussion

Immunohistochemistry assays of tumour biopsies to evaluate immune checkpoint target expression have recently been implemented in the clinical routine to select patients eligible for immune checkpoint inhibition. However, it is becoming increasingly clear that immune checkpoint targets are highly dynamic and a better understanding of the spatiotemporal dynamics of the tumour-immune microenvironment, which is difficult to obtain with a single biopsy, is critical for effective therapies to be developed and clinically applied [20].

In the present study, we evaluated the ability of ^89^Zr-DFO-6E11 to visualize and quantify the therapy-induced changes in PD-L1 expression following radiotherapy and the predictive value of ^89^Zr-DFO-6E11 PD-L1 PET prior to immune checkpoint blockade of PD-L1. Due to the high non-tumour (i.e., lymphoid tissue) expression of PD-L1, we initially sought to define the optimal antibody dose by decreasing the uptake in endogenous tissue—a common optimization approach in PET imaging studies. Increasing the administered antibody dose to 30 μg decreased the splenic uptake from 14.44 ± 3.10 to 5.59 ± 0.51 %ID/g and concurrently allowed visualisation of tumour PD-L1 by increasing the tumour uptake ~ 5-fold from 0.35 ± 0.04 to 1.72 ± 0.24 %ID/g. Additionally, the non-specific liver uptake decreased with increasing dose confirming this organ as the major site of clearance of 6E11. PD-L1 is not a traditional tumour imaging target as the expression is higher in non-tumour tissues, and it can be argued that it is not desired to block lymphoid uptake completely as ^89^Zr-DFO-6E11 uptake in these tissues might serve as a surrogate for activation state of the body’s immune system. In addition, choosing a dose that saturates the antigen sink in lymphoid tissue completely might tip the balance in another individual and potentially block tumour target. Indeed, it has been suggested that low specific activity ^89^Zr-DFO-6E11 may be needed to accurately determine tumour PD-L1 expression levels [21].

In vivo imaging confirmed a similar distribution pattern with highest ^89^Zr-DFO-6E11 uptake in lymph nodes and spleen in HCC827 tumour-bearing mice. Gamma counting of the spleen after the last imaging time-point was higher than the uptake measured by PET ROI analysis (~ 14 ex vivo vs. 6.5 in vivo %ID/g) most likely due to limited image contrast and the partial volume effect introduced by PET ROI quantification of the spleen. The mean and maximum tumour uptake differed substantially, indicative of a relatively heterogeneous tumour uptake of ^89^Zr-DFO-6E11. No further increase in tumour-to-background ratios was observed beyond 72 h, which also has been confirmed as optimal imaging time-point for other atezolizumab-based tracers [14, 15]. Overall, the tumour uptake 144 h post-injection found in the longitudinal imaging study (Fig. 2) was approximately 2-fold higher than observed for the dose escalation study (Table 2) with a 30-μg co-dose. This discrepancy can be explained by the shift in antibody conjugation strategy to site-specifically labelled ^89^Zr-DFO-6E11. As evident from Table 1, site-specific ^89^Zr-DFO-6E11 was presented with higher immuno-reactivity, affinity, and stability—factors known to influence target binding and accumulation.

Our in vivo PET imaging and biodistribution studies in five different mouse models of humane and murine cancer clearly demonstrated the PD-L1 binding specificity of ^89^Zr-DFO-6E11, where the ^89^Zr-DFO-6E11 tumour uptake was consistent with both in vitro and ex vivo findings. In general, the models applied in this study were considered to be well suited. Firstly, the cell lines used express endogenous levels of PD-L1 and are not engineered to express high, constitutive levels of PD-L1 as the frequently used hCHO-PD-L1 cell line. Secondly, due to the cross-reactivity of ^89^Zr-DFO-6E11, background levels are present, making it easier to compare preclinical and clinical data. In the present study, the splenic PET uptake did not differ between NMRI nude (8.2 ± 1.0 %ID/g, Fig. 2b) and BALB/C mice (8.46 ± 0.48 %ID/g, data not shown) 72 h post-injection. Thus, the background levels can be considered comparable, and the ^89^Zr-DFO-6E11 tumour uptake was compared across mouse strains. Evident from the flow cytometric and IHC analysis of H1703, this cell line can be considered PD-L1 negative, and the non-specific ~ 1.5 %ID/g tumour uptake attributed to the enhanced permeability and retention effect. The specificity for tumour PD-L1 was also confirmed by the dose escalation study, where a blocking tendency was observed with increasing 6E11 co-dose. Even though tumour uptake appeared high in HCC827 tumour-bearing mice co-injected with 500 μg, this was expected as the endogenous PD-L1 levels are blocked allowing for more tracer in the circulation available for tumour accumulation. The tumour-to-background ratios, however, witnessed that the tumour-to-muscle and tumour-to-blood ratio decreased from 17.23 ± 3.09 to 10.34 ± 3.50 and 14.05 ± 4.70 to 1.80 ± 0.33, from a 100- to a 500-μg co-dose, respectively.

External radiation therapy is well known to upregulate tumour PD-L1 expression levels, partially as a part of an immunogenic anti-tumour immune response, and also as a resistance mechanism to facilitate immuno-suppression and tumour relapse [22–25]. For that reason, there is an increasing interest in combining radiotherapy and immune checkpoint blockade to synergistically improve therapeutic efficacy. We observed an increased ^89^Zr-DFO-6E11 uptake in the spleens and tumours of irradiated CT26 tumour-bearing mice, possibly originating from a combination of increased expression levels and increased infiltration of PD-L1 positive cells due to the inflammatory state. Noteworthy is that ^89^Zr-DFO-6E11 accurately detected the peripheral changes in PD-L1 levels induced by local radiotherapy with increased splenic uptake. This finding further highlights the potential of PET-based methods for in vivo biomarker assessment on a whole-body, non-invasive level and the clinical utility of ^89^Zr-DFO-6E11 as an indicator of immune system activity.

The utility of antibody-based PD-L1 PET in irradiated NSCLC tumours has also been investigated by Ehlerding et al. [15], who also demonstrated a significant increase in tumour uptake of ^89^Zr-DFO-atezolizumab after radiotherapy. The uptake levels cannot be directly compared due to different models, injected doses, and therapy regimen, but the significant ~ 1.5-fold increase in tumour-to-muscle ratios of irradiated mice 72 h post-injection is equal to that obtained in this study (20.68/14.09, 1.48-fold increase). Furthermore, Ehlerding and colleagues showed that the source of PD-L1 originated from CD45^+^ as well as CD45^−^ negative cells in the NSCLC tumours. The expression of target on antigen-presenting cells highlights the need for a biologically inert imaging agent giving rise to concerns regarding the use of a full-length antibody tracer for visualizing target expression. However, the Fc engineering of atezolizumab aids in silencing the effector functions merely leading to neutralization of receptors [26], thus supporting the usage of atezolizumab-based tracers. Together, with the different read-outs from available clinical companion diagnostic assays for anti-PD-L1 therapy in mind [27, 28], this encourages the practice of imaging with a radiolabelled version of the therapeutic antibody enabling quantification of exact distribution pattern within the body and the delivery to tumour prior to and during therapy.

Major questions arise in the search for a predictive biomarker of response to immune checkpoint therapy. Indeed, it can be questioned whether tumour PD-L1 is the most predictive biomarker since clinical responses have been reported among ~ 15% of patients with PD-L1 negative tumours shown by IHC [29]. Is the “failure” rate of prediction with the approved diagnostic assays due to limited sampling or to the unanswered facets of PD-L1 biology, and in the latter case, is it then too simple merely showing PD-L1 biology? Regardless of the cause, our data indicate that ^89^Zr-DFO-6E11 can be used as a companion diagnostic method to select patients eligible for PD-L1 blockade, which recently have been backed up by clinical data [17]. We found the maximum tumour-to-muscle ratios of ^89^Zr-DFO-6E11 CT26 tumour-bearing mice to correlate with response to anti-PD-L1 therapy alone or in combination with radiotherapy. No association with the mean tumour-to-muscle ratio was found. This is agreeing well with the fact that maximum values of tumour imaging markers are considered more robust indicators of prognosis and are therefore the most commonly utilized measure in the clinic [30–32]. Together with the prominent expression of PD-L1 on antigen-presenting cells and the heterogenous distribution, e.g., migration and clustering, of these within a tumour, this could explain the superiority of the maximum tumour-to-muscle ratio of ^89^Zr-DFO-6E11 for response prediction. To our knowledge, this is the first study correlating response to immunotherapy with pre-therapy PD-L1 PET imaging in a preclinical mouse model demonstrating its clinical potential. Furthermore, we utilize a site-specific conjugation methodology resulting in uniform, well-defined conjugates with minimal loss of immuno-reactivity and high reproducibility. Together, this highlights the enthusiasm for further clinical translation of site-specific ^89^Zr-DFO-6E11. However, the results obtained herein may not be generally applicable to all cancer types, models, and treatments. Also, to determine an objective and reproducible method, i.e., cutoff for PET tumour uptake or tumour-to-muscle ratio, is critical for accurate stratification of patients in future clinical studies. Future preclinical studies investigating multiple tumour types, dosing regimens, and scan protocols will elucidate whether ^89^Zr-DFO-6E11 is a true predictor of response to PD-L1 immune checkpoint blockade.

## Conclusion

Cancer therapeutics is progressively moving away from compounds that target tumours broadly. Modulating immune responses by blockade of immune checkpoints, and indeed, the dynamic nature of these, is beginning to be realized. We herein show that ^89^Zr-DFO-6E11 PET specifically detects various degrees of PD-L1 expression levels at baseline and after radiotherapy in mouse models of murine and human cancer. Importantly, we demonstrated that the tumour-to-muscle ratio of ^89^Zr-DFO-6E11 PET in this experimental setup was predictive of response to PD-L1 immune checkpoint inhibition in a syngeneic mouse tumour model. Moreover, ^89^Zr-DFO-6E11 PET might serve as an early identifier of immune response activation in a clinical setting, warranting the further clinical development to aid in determining proper therapies and monitoring patient responses.

## Electronic supplementary material

Fig. S1 **Tumour volume of treatment groups at day 4.** There was no difference in tumour volumes between the control, XRT, XRT + anti-PD-L1 and anti-PD-L1 treatment groups at day 4 (*N* = 8/group). Data are presented as mean ± SEM. *ns = no significance.* XRT = external radiation therapy. (JPG 1911 kb)

Fig. S2 **Representative PET/CT images illustrating ROI analysis of the tumour and muscle uptake in anti-PD-L1 treated CT26 tumour-bearing mice.** (a) Coronal PET/CT image 72 hours post-injection of ^89^Zr-DFO-6E11. Muscle ROI indicated by arrow. (b) (Axial PET/CT image 72 hours post-injection of ^89^Zr-DFO-6E11. Tumour ROI indicated by arrow. (JPG 557 kb)

Fig. S3 **Correlation plots of % tumour growth increase at various time-points following therapy initiation and the**^**89**^**Zr-DFO-6E11 tumour-to-muscle ratio.** Tumour growth increase from day 4 to day 15 (a), 19 (c) and 22 (e) expressed as % compared to the tumour(mean)/muscle(mean) ratio of ^89^Zr-DFO-6E11 in mice treated with 10 mg/kg anti-PD-L1 (*N*=16). Tumour growth increase from day 4 to day 15 (b), 19 (d) and 22 (f) expressed as % compared to the tumour (max)/muscle(mean) ratio of ^89^Zr-DFO-6E11 in mice treated with 10 mg/kg anti-PD-L1 (N=16). (JPG 294 kb)

ESM 4 (DOCX 73 kb)
