# Supplementary figures and images for: Quantitative PET imaging of PD-L1 expression in xenograft and syngeneic tumour models using a site-specifically labelled PD-L1 antibody
Source: Eur J Nucl Med Mol Imaging. 2019 Dec 27;47(5):1302–13. doi: 10.1007/s00259-019-04646-4 (PMC7101303; doi:10.1007/s00259-019-04646-4)

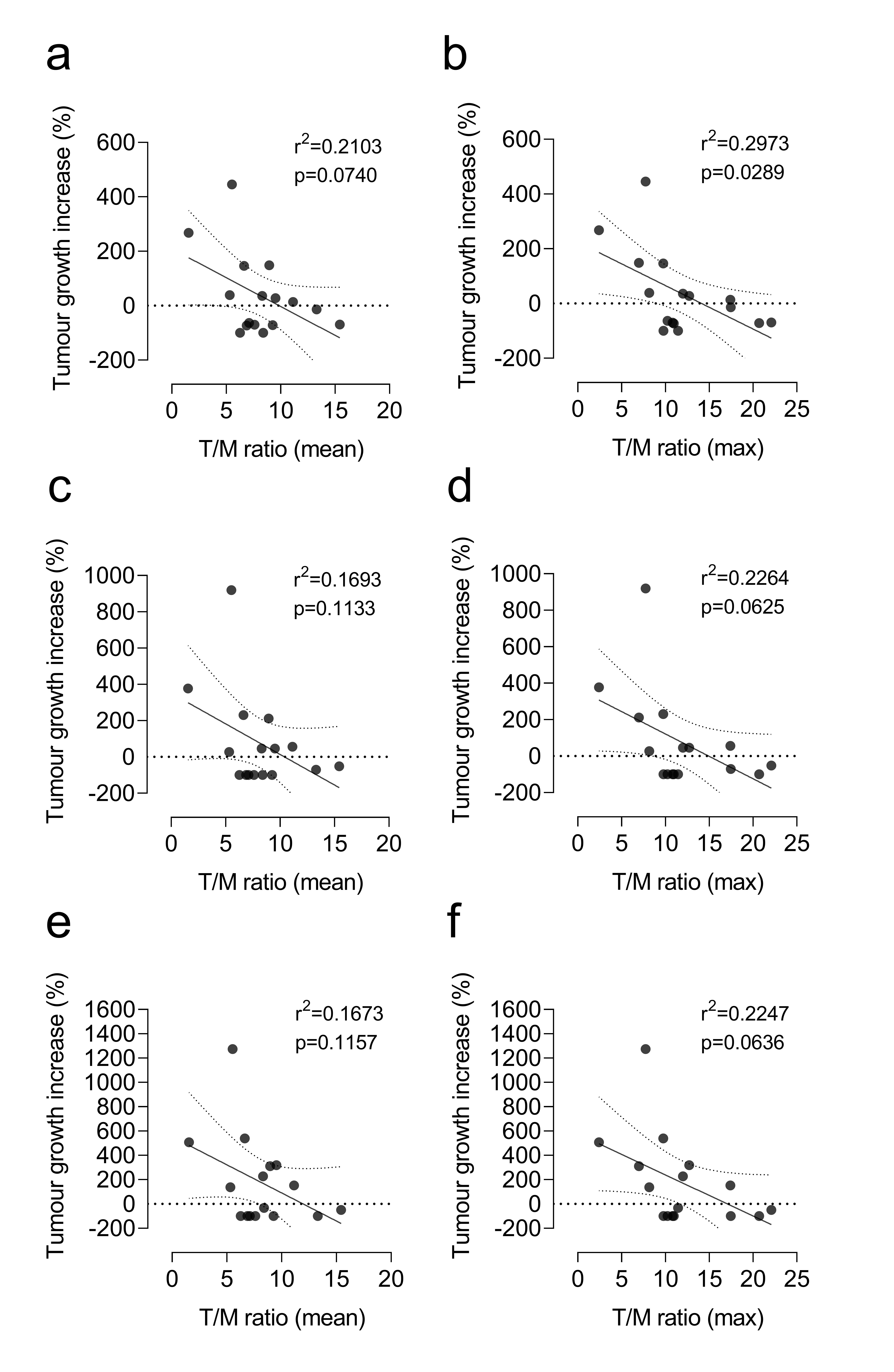

Supplement: Supplementary file 1 — Tumour volume of treatment groups at day 4. There was no difference in tumour volumes between the control, XRT, XRT + anti-PD-L1 and anti-PD-L1 treatment groups at day 4 (N = 8/group). Data are presented as mean ± SEM. ns = no significance. XRT = external radiation therapy. (JPG 1911 kb) [file 259_2019_4646_MOESM1_ESM.jpg]

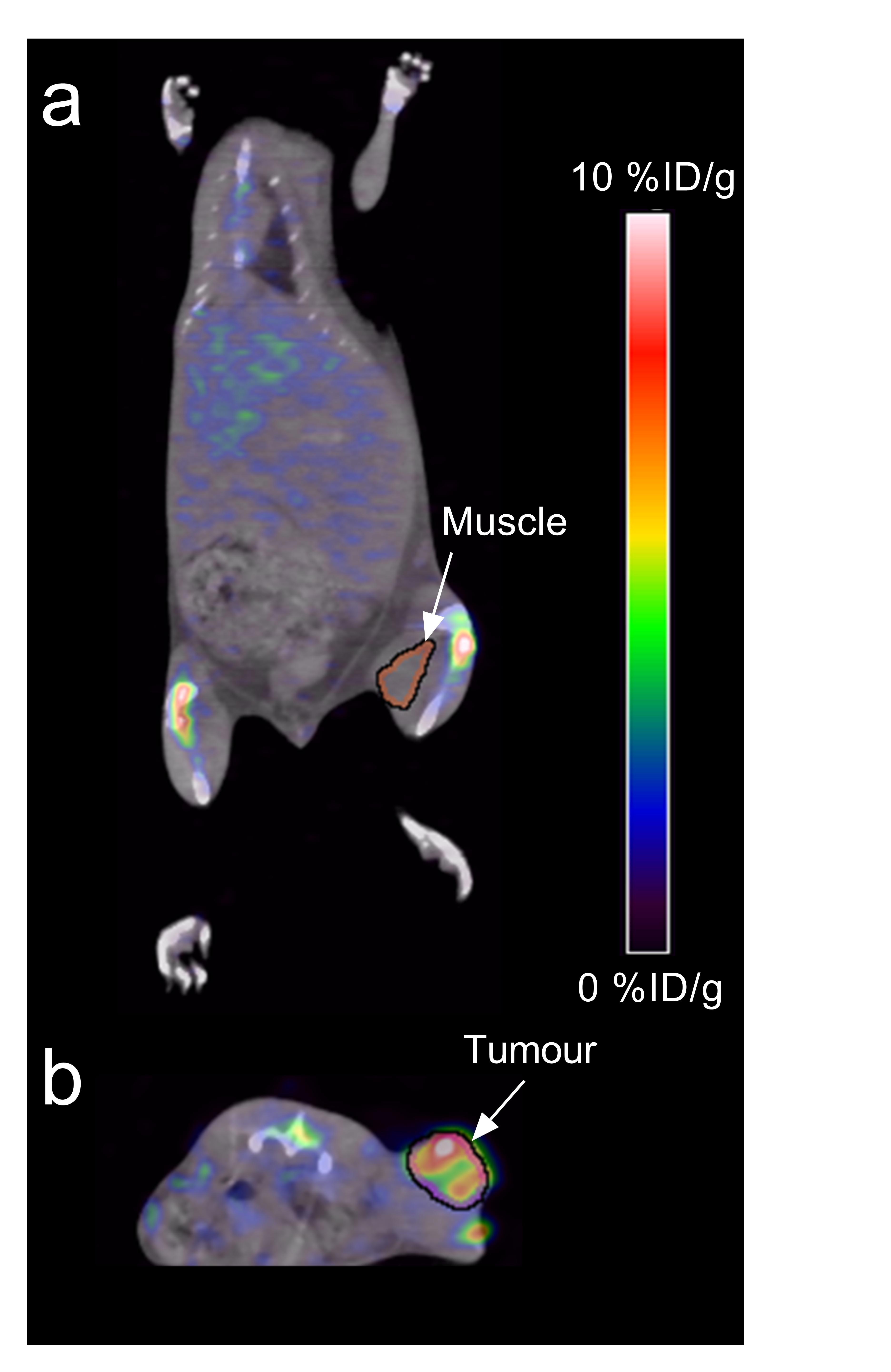

Supplement: Supplementary file 2 — Representative PET/CT images illustrating ROI analysis of the tumour and muscle uptake in anti-PD-L1 treated CT26 tumour-bearing mice. (a) Coronal PET/CT image 72 hours post-injection of 89Zr-DFO-6E11. Muscle ROI indicated by arrow. (b) (Axial PET/CT image 72 hours post-injection of 89Zr-DFO-6E11. Tumour ROI indicated by arrow. (JPG 557 kb) [file 259_2019_4646_MOESM2_ESM.jpg]

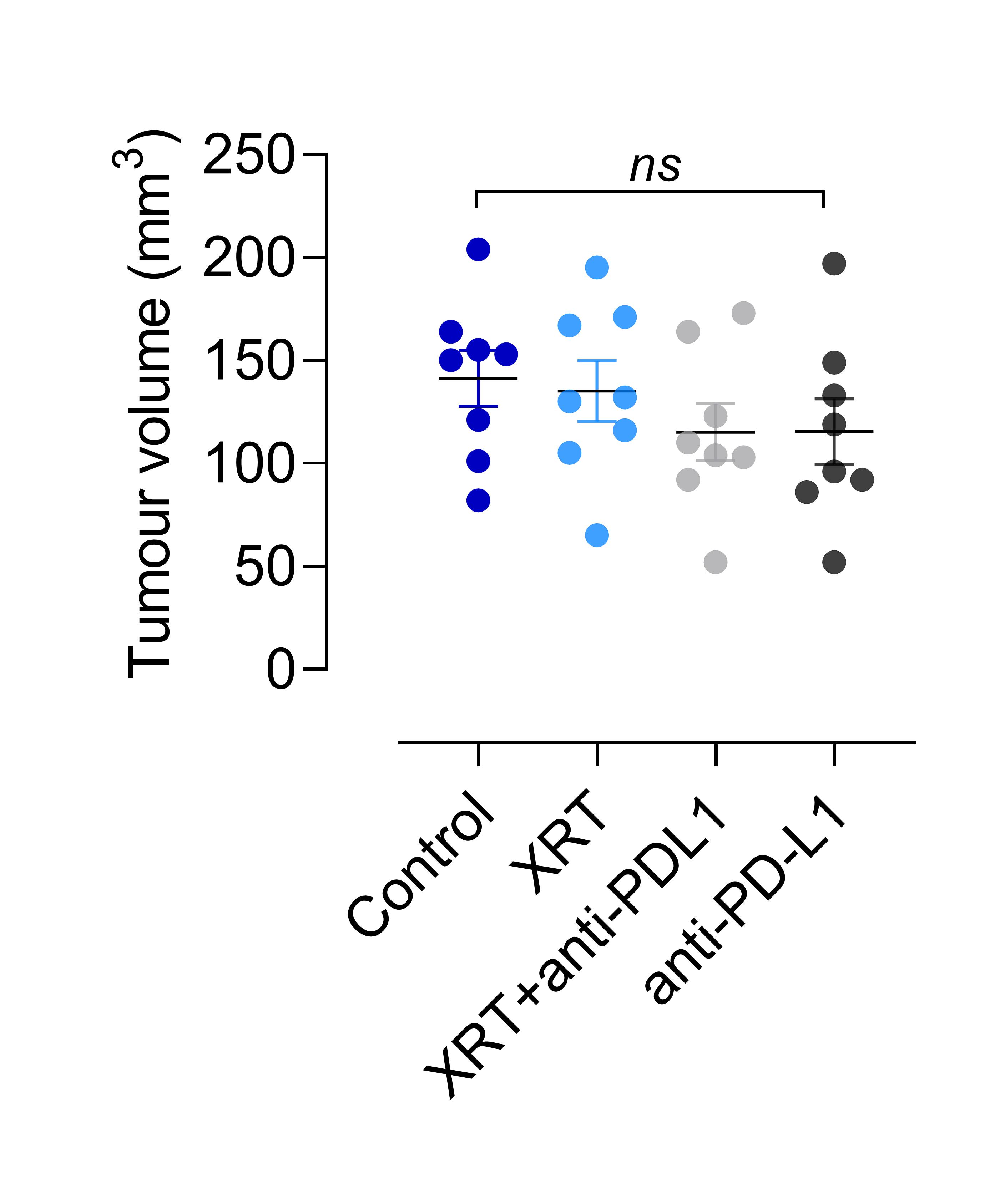

Supplement: Supplementary file 3 — Correlation plots of % tumour growth increase at various time-points following therapy initiation and the89Zr-DFO-6E11 tumour-to-muscle ratio. Tumour growth increase from day 4 to day 15 (a), 19 (c) and 22 (e) expressed as % compared to the tumour(mean)/muscle(mean) ratio of 89Zr-DFO-6E11 in mice treated with 10 mg/kg anti-PD-L1 (N=16). Tumour growth increase from day 4 to day 15 (b), 19 (d) and 22 (f) expressed as % compared to the tumour (max)/muscle(mean) ratio of 89Zr-DFO-6E11 in mice treated with 10 mg/kg anti-PD-L1 (N=16). (JPG 294 kb) [file 259_2019_4646_MOESM3_ESM.jpg]
