# Supplementary material for: Quantitative PET imaging of PD-L1 expression in xenograft and syngeneic tumour models using a site-specifically labelled PD-L1 antibody
Source: Eur J Nucl Med Mol Imaging. 2019 Dec 27;47(5):1302–13. doi: 10.1007/s00259-019-04646-4 (PMC7101303; doi:10.1007/s00259-019-04646-4)
Supplement: Supplementary file 4 — (DOCX 73 kb) [file 259_2019_4646_MOESM4_ESM.docx]

# Supplementary

**Table S1:** Buffer and plasma stability of ^89^Zr-DFO-6E11 evaluated by size exclusion-high performance liquid chromatography (SEC-HPLC) and radio-thin layer chromatography (radio-TLC) 24, 72 and 144 hours after end-of-synthesis (EOS). Data are expressed as % intact tracer.

|  | Buffer (HPLC) | Buffer (TLC) | Plasma (HPLC) | Plasma (TLC) |
| --- | --- | --- | --- | --- |
| EOS (0 h) | 97.7%   \| **97.5 %** \| \| --- \| \| 92.3 % \| \| 83.8 % \| \| 72.0 % \| | >99% | - | - |
| 24 h | 92.3% | >99% | 50.8% | >99% |
| 72 h | 83.8% | >99% | 50.9% | >99% |
| 144 h | 72.0% | >99% | 47.8% | >99% |
